# Cook County Hospital

**Published:** 1866-02

**Authors:** 


### COOK COUNTY HOSPITAL.

The Cook County Hospital was opened to the public on the tenth of January. The building has been cleansed and refitted throughout, so that it offers accommodation equal to any other charitable hospital in the country. In addition to the public wards which are designed for the reception of the pauper patients of the city and county, there are suits of apartments prepared for the use of patients who desire to enjoy the advantages of hospital treatment without sacrificing the privacy of home. Judging from the number of applications for accommodation of the kind, this will prove to be for the public one of the most attractive features of the institution.

The wards of the hospital will be open to medical students, and to all regular practitioners of medicine, on Tuesday and Friday of each week throughout the year, at half-past one o'clock P. M. From the first of October to the last of May, a medical and a surgical clinic will be held on each of the days above mentioned. During the remaining four months of the year there will be a medical clinic every Tuesday, and a surgical clinic every Friday. As far as possible the *post mortem* inspection of the dead will be made in the presence of those who desire to follow the practice of the hospital, and no effort will be spared to render available the materials which may be furnished for the study of pathological anatomy.

The immediate care of the patients is confided to a resident physician and his assistant, who constitute, under the direction and supervision of the visiting physicians and surgeons, the house-staff of the hospital. An examination will be conducted every spring and fall by the Medical Board of the hospital, at which time appointments to fill the vacancies which will occur in the house-staff, through the expiration of the terms of service of its several members, will be conferred upon the most deserving of the applicants for position in the hospital. As the examination is open to all who choose to apply, and as the awards of the examiners are based wholly upon the merits of the candidates, this arrangement affords a rare opportunity for young men who desire to supplement their theoretical knowledge by a practical introduction to the labors of the profession under circumstances the most favorable to the acquisition of experience.

The recent examination resulted in the selection of N. I. Quales, M. D., a member of the class recently graduated at the Rush Medical College, for the position of resident House Physician to the hospital during a term of one year from Feb. 1st, 1866. Mr. W. H. Hutchinson, of the junior class at the Chicago Medical College, received the appointment of Assistant House Physician for the same period of time. As these appointments are based solely upon the merits of the candidates as determined by a strict and impartial examination before a committee of the Medical Board of the hospital, they constitute the highest prize that is afforded for the competition of the medical students who gather in this city.

